# Meningococcal carriage and transmission dynamics in college students in Louisville, Kentucky

**DOI:** 10.1371/journal.pone.0344194

**Published:** 2026-03-05

**Authors:** Forest W. Arnold, Leslie Wolf Parrish, Subathra Marimuthu, Jamie Findlow, Angela Quinn, Vidyulata Salunkhe, Daniya Sheikh, Phillip Bressoud, T’shura Ali, Dawn Balcom, Mohammad Ali, Ryan S. Doster, Deepti Deepti, Mohammad Tahboub, Fama Ndiaye, Jay Lucidarme, Stephen A. Clark, Ray Borrow, Paul Balmer, Steven Gootee, for the CERID study group

**Affiliations:** 1 Division of Infectious Diseases, School of Medicine, University of Louisville, Louisville, Kentucky, United States of America; 2 Global Vaccines, Medical Affairs, Pfizer Ltd, Tadworth, Surrey, United Kingdom; 3 Medical Affairs, Pfizer Inc, Collegeville, Pennsylvania, United States of America; 4 Department of Internal Medicine, Campus Health Services, University of Louisville, Louisville, Kentucky, United States of America; 5 Department of Epidemiology and Population Health, School of Public Health and Information Sciences, University of Louisville, Louisville, United States of America; 6 Medical Affairs and Evidence Generation, Pfizer Inc, Collegeville, Pennsylvania, United States of America; 7 Department of Microbiology and Immunology, University of Louisville School of Medicine, Louisville, Kentucky, United States of America; 8 Meningococcal Reference Unit, United Kingdom Health Security Agency, Manchester Royal Infirmary, Manchester, United Kingdom; Defense Threat Reduction Agency, UNITED STATES OF AMERICA

## Abstract

**Background:**

*Neisseria meningitidis* is a cause of meningitis and outbreaks of it among young adults, especially college students. Rates of nasopharyngeal colonization and prevalence of specific capsular groups vary with age, geography as well as time, and may be influenced by meningococcal vaccination. The objective of this study was to measure the change in colonization rate, and define which meningococcal genogroups were present, in college students over a 3-month semester.

**Methods:**

This was a prospective, longitudinal cohort study with sequential oropharyngeal swabbing among college students at the University of Louisville (UofL) in Louisville, Kentucky from August to November 2022. Participants were ≥18 years of age and were enrolled within 48 hours of moving to campus-affiliated housing. Oropharyngeal swabs were collected at enrollment, one month and at three months. Samples were screened for *N. meningitidis*, and isolates were characterized using phenotypic and genotypic methods. Behavior questionnaires were obtained at each visit to identify risk factors for *N. meningitidis* colonization.

**Results:**

A total of 1047 participants were seen initially, of whom 821 attended all three visits. The baseline colonization rate was 3.5% followed by 3.9% after one month and 5.7% after three months. The genogroups of recovered isolates were capsule null (48%), B (38%; of which 11% were expressing capsule) and E (12%). No genogroup ACWY isolates were recovered. A total of 36% of participants had a history of receiving at least one MenB vaccine dose and 74% had a history of receiving at least one MenACWY vaccine. Risk factors for *N. meningitidis* nasopharyngeal carriage included being a second-year student, living on campus for the second year, smoking/vaping, kissing and sexual contact.

**Conclusions:**

An increase in *N. meningitidis* colonization over the 3-month semester was observed from 3.5% to 5.7%. The overall proportion of student carriers was significantly lower, and there were no genogroup A, C, W or Y strains isolated compared to studies conducted prior to the availability of meningococcal vaccines and the COVID-19 pandemic. However, genogroup B carriage, transmission and acquisition were almost identical to pre-COVID pandemic studies. This study reinforces the importance of periodic epidemiological monitoring of carriage as well as disease.

## Introduction

*Neisseria meningitidis* is a significant public health threat because of its ability to asymptomatically colonize the nasopharynx of individuals and potentially cause invasive meningococcal disease. Carriers may inadvertently transmit *N. meningitidis* through various means including aerosols, droplets, and activities that involve saliva exchange, such as kissing or sharing utensils [1,2]. Carriage rates vary with age, with a notable increase during adolescence that peaks in young adults, typically around 19 years of age [3]. This increase is attributed to changes in social behaviors including living in close quarters, such as dormitories, and engagement in high-risk behaviors (*e.g.*, close social contact) that facilitate transmission and exposure [4,5].

Globally, serogroups A, B, C, W, and Y have historically been responsible for most invasive meningococcal disease cases as well as outbreaks, and are preventable with vaccines [6]. Serogroup X has also recently been responsible for outbreaks of disease in Africa, and is now also preventable through immunization. In the US, the first conjugate meningococcal serogroup A, C, W and Y (MenACWY) vaccine was licensed in 2005, and currently, two MenACWY conjugate vaccines are widely available in the US. Adolescents 11–12 years of age are currently recommended to routinely receive a MenACWY vaccine, with a booster dose at 16 years old [7]. Two recombinant serogroup B (MenB) vaccines were licensed in 2014 and 2015. These vaccines are recommended on a shared clinical decision-making basis for those age 16–23 years with a preferred age of 16–18 years. Separate immunization recommendations exist for immunosuppressed populations [8]. The first pentavalent meningococcal ABCWY vaccine was licensed in 2023 and is currently recommended for those >10 years who require immunization against MenACWY and MenB at the same visit.

Despite the recognized risk of meningococcal disease and prior studies of *N. meningitidis* carriage among college students from other countries [5,9,10], in the post-COVID pandemic period, data regarding *N. meningitidis* carriage in the US population is needed to inform vaccination strategies and public health efforts. In many countries, COVID-19 pandemic prevention control measures, including restrictions on social interactions, had a significant impact on disease rates and may have also impacted carriage rates, particularly in college populations [11]. Understanding of the current prevalence of *N. meningitidis* carriage, carriage of specific genogroups, and rates of new acquisitions is needed to inform public health strategies to mitigate the risk of meningococcal outbreaks in college settings. The primary objective of this study was to investigate sequential changes in *N. meningitidis* carriage rates among college students living on campus over a 3-month semester. The secondary objective of this study was to identify demographics, social behaviors, and vaccination status associated with meningococcal carriage prevalence and acquisition.

## Materials and methods

### Study design and population

This was a prospective, longitudinal cohort study with sequential oropharyngeal swabbing among University of Louisville (UofL) college students in Louisville, Kentucky. Students were recruited from August 2−23, 2022 during the fall semester (August through November 2022) of the 2022−23 academic year. This was a descriptive study designed to determine meningococcal carriage, so no *a priori* sample size or power calculation was performed. Instead, participants were recruited using a convenience sample, and all eligible students available during the study period were included to provide a comprehensive characterization of the data.

The study population included currently enrolled UofL college students, regardless of underlying diseases or meningococcal vaccination status. Inclusion criteria included students residing in campus affiliated housing, being at least age 18 years, and being able to provide an oropharyngeal sample within 48 hours of arrival to campus, and during the subsequent sampling windows. Students who arrived on campus outside of “move-in week” were not considered eligible to enroll in the study. Participants were asked to complete a total of three study visits. The first study visit occurred within 48 hours of arriving on UofL campus for the academic semester. The second study visit occurred approximately four weeks after the first swab was obtained (±4 days), and the third study visit occurred approximately 12 weeks after the first swab was obtained (±1 week). Informed consent was obtained from all subjects involved in the study electronically via REDCap electronic data capture hosted at the University of Louisville e-consent function [12,13]. This study was approved by the University of Louisville Institutional Review Board (IRB# 22.0474)

### Study procedures

After informed consent was obtained, trained research coordinators collected an oropharyngeal swab and administered a questionnaire (Supplement) at each study visit that included demographics, meningococcal vaccination information, behavioral patterns including known risk factors for meningococcal carriage (e.g., smoked tobacco/cigarettes, nicotine e-cigarette, cannabis, attended a party, kissed or had sexual contact (“hooked up”) within the last week). ‘At-risk’ was defined as having at least one of the aforementioned risk factors within the week prior to enrollment while ‘low risk’ was defined as not having any of the factors in that time period. Campus affiliated housing was defined as dormitory (dormitory or fraternity/sorority) or non-dormitory (house or apartment).

### Clinical and Laboratory procedures

Swabbing was performed using a standardized and methodological approach. A sterile flocculated swab was wiped across the posterior oropharynx from one tonsillar area to the other and the swab placed immediately in 2 mL of skim milk/tryptone/glucose/glycerol (STGG) transport media [14]. Swabs in STGG media were maintained at 4ºC from the time of sample collection, transported to the laboratory and immediately processed in a certified Class II Biosafety Cabinet. After vortexing each sample, a 1 mL aliquot of media was transferred into each of 2 labelled cryovials (VWR International, Radnor, PA, US). Samples were then frozen at −80ºC within 8 hours of collection. Aliquots were stored at −80ºC in two separate boxes so that two separate shipments could be made to the reference laboratory.

Aliquots were sent frozen on dry ice to the Meningococcal Reference Unit (MRU), United Kingdom Health Security Agency (UKHSA), in Manchester, United Kingdom. Samples (100 µL) were screened for *N. meningitidis* using GC Selective Agar with VCAT (Vancomycin, Colistin, Amphotericin B & Trimethoprim, Oxoid, UK). Suspected meningococcal colonies were tested using Oxidase test and Gram’s stain. Oxidase-positive, Gram-negative diplococci were stored for further characterization.

Genome sequence analysis (2 x 300 bp paired end) was performed on all oxidase-positive, Gram-negative diplococci using an Illumina platform (MiSeq or NextSeq 1000). Genomic DNA was extracted using MAG-Bind Bacterial DNA kits (Omega Bio-tek, Inc., GA, USA). Library preparation was performed using Illumina DNA Prep kits (Illumina, CA, USA). Draft genomes were assembled using Shovill (v1.1.0; with minimum contig length = 200 and minimum contig coverage = 10) [15,16]. Assembled genomes were uploaded to PubMLST.org/Neisseria for gene indexing [17]. All genogroup A, B, C, W or Y meningococcal isolates were then serogrouped using a Dot-Blot ELISA as previously described [18].

### Statistical analysis

The primary endpoint was the prevalence of meningococcal carriage in this study population. The numerator for the calculation was those with *N. meningitidis* carriage, and the denominator was the participants with a valid result from their oropharyngeal samples. The prevalence was calculated for each genogroup and overall (all genogroups combined), as well as for each visit and overall (all three visits combined). The changes in the carriage status in those carrying meningococci at visits 1 and/or 2 were compared with the subsequent visit and reported using frequency and percentage. Where meningococcal carriage was detected at both comparative visits, whole genome sequence derived data including genogroup, factor H binding protein (fHbp) peptide, Neisserial Heparin Binding Antigen (NHBA) peptide, *Neisseria* Adhesin A (NadA) peptide, Porin A (PorA) subtype, sequence type (ST) and clonal complex (CC) were used to determine if it was the same meningococcal strain or a different strain.

Participant characteristics were summarized as frequency and percentage (%) for categorical variables and mean and standard deviation (SD) for continuous variables. Bivariate and multivariable logistic regression models were used to examine the association of demographic, geographic location, behavioral characteristics, and vaccinated status with the meningococcal carriage prevalence. Only the variables associated with the outcome at *P* < 0.10 in the bivariate models were included in the multivariable model. Crude and adjusted odd ratios (OR) along with 95% confidence intervals (CIs) were reported. Firth’s Penalized Likelihood estimation under the logistic model was used in the event of small sample sizes. The analysis was performed by visit and for all three visits combined. Statistical significance was set at p-value <0.05. All analyses were performed using SAS Studio Release: 3.81 (Enterprise Edition), SAS Institute Inc., Cary, NC, USA.

## Results

**Fig 1:**
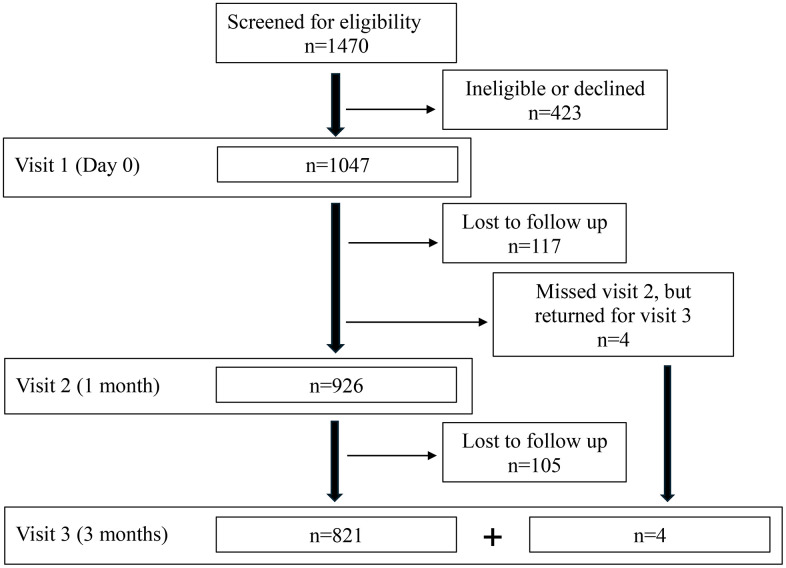
Flowchart of study population on campus at each visit who were enrolled and those who presented for a follow-up visit.

A total of 1047 students were enrolled and swabbed at visit 1, 926 were swabbed at visit 2 and 825 were swabbed at visit 3 (Fig 1). A total of 821 students provided all three swabs; of those, the mean age was 19.1 years, 40% were males, 21% were African American, 7% were of Asian race and 6% were of Hispanic ethnicity (Table 1). Most participants that provided all three swabs were in the first year of college (73%), were living on campus for the first time (75%), and most were living in the schools’ dormitories (77%) (Table 1). The proportion of participants ‘at risk’ for *N. meningitidis* carriage increased across each of the three visits, respectively (60% vs 69% vs 72%) (Table 2). Of the 821 students who went to all 3 visits and were swabbed, 299 (36%) received at least one dose of MenB vaccine, whereas 611 (74%) received at least one dose of a MenACWY vaccine. (Table 3).

**Table 1:** The Socio-demographic characteristics for the study participants. All values are listed as n (%) unless otherwise noted.

Characteristics	All Participants n = 1047 (%)^a^	Participants present at all 3 visits n = 821 (%)^b^
Age (years) – mean (SD)	19.1 (1.5)	19.1 (1.6)
**Sex** ^**a**^		
Male	423 (40)	329 (40)
Female	623 (60)	491 (60)
Missing	1 (0.1)	1 (0.1)
**Race**		
Asian	54 (5.1)	47 (5.7)
African American	171 (16)	136 (16)
White	711 (68)	552 (67)
Mixed/Other	110 (11))	86 (10))
Missing	1 (0.1)	0
**Ethnicity**		
Hispanic	67 (6.4)	51 (6.2)
Non-Hispanic	902 (86)	718 (88)
Not specific	78 (7.4)	52 (6.3)
Number of roommates – mean (SD)	1.6 (5.2)	1.7 (5.8)
**Student Status**		
1st Year	775 (74)	597 (73)
2nd Year	179 (17)	150 (18)
3rd Year	63 (6)	49 (6)
4th Year	23 (2.2)	19 (2.3)
5th Year or higher	7 (0.7)	6 (0.7)
**Living in campus-affiliated housing**		
First time	801 (77)	619 (75)
Second time	179 (17)	148 (18)
Third time	53 (5.1)	42 (5.1)
Other	14 (1.3)	12 (1.5)
**Residential situation**		
Dorm	831 (79)	635 (77)
Non-dorm	216 (21)	186 (23)
**Other cohort identification**		
Member of sport team	39 (3.7)	25 (3)
Member of club team (Ex: chess club)	50 (4.8)	41 (5)
Member of a band	40 (3.8)	37 (4.5)
Not applicable	774 (74)	605 (74)
Missing	144 (14)	113 (14)

^a^Sex at birth

^b^Values >10 were rounded to the nearest whole percentage.

**Table 2:** The behavioral characteristics among the study participants present for all three visits; (n = 821).

	Visit 1 n(%)^a^	Visit 2 n (%)^a^	Visit 3 n (%)^a^
**Having a cold, sore throat, or respiratory infection**			
Yes	22 (2.7)	75 (9.1)	123 (15)
No	797 (97)	743 (91)	698 (85)
Missing	2 (0.2)	3 (0.4)	0 (0)
Antibiotic Intake			
None in the last month	738 (90)	726 (88)	697 (85)
Stopped in last month	38 (4.6)	35 (4.3)	44 (5.4)
Stopped in last week	13 (1.6)	24 (2.9)	35 (4.3)
Yes, currently taking	30 (3.7)	33 (4.0)	45 (5.5)
Missing	2 (0.2)	3 (0.4)	0 (0)
**Smoked tobacco/cigarettes in the last week**			
Yes	31 (3.8)	47 (5.7)	63 (7.7)
No	788 (96)	771 (94)	757 (92)
Missing	2 (0.2)	3 (0.4)	1 (0.1)
**Smoked nicotine e-cigarette (vape) in the last week**			
Yes	117 (14)	140 (17)	161(20)
No	702 (86)	678 (83)	660 (81)
Missing	2 (0.2)	3 (0.4)	0 (0)
**Smoked pot (marijuana, weed, grass, etc.) in the last week**			
Yes	103 (13)	145 (18)	168 (21)
No	715 (87)	673 (82)	653 (80)
Missing	3 (0.4)	3 (0.4)	0 (0)
**If yes, how?**			
Bong	16 (2)	18 (2.2)	21 (2.6)
Bowl	10 (1.2)	12 (1.5)	18 (2.2)
Joint	35 (4.3)	54 (6.6)	66 (8)
Vape	38 (4.6)	53 (6.5)	55 (6.7)
Other	4 (0.5)	8 (1)	8 (1)
**Smoked by sharing a device in the last week**			
Yes	33 (4)	48 (5.9)	63 (7.7)
No	786 (96)	770 (94)	758 (92)
Missing	2 (0.2)	3 (0.4)	0 (0)
**No. of times been to a party in the last week**			
0	638 (78)	423 (52)	444 (54)
1	128 (16)	215 (26)	174 (21)
2	39 (4.8)	111 (14)	116 (14)
3+	14 (1.7)	69 (8.4)	87 (11)
Missing	2 (0.2)	3 (0.4)	0 (0)
**No. of people kissed or hooked up^b^ with in the last week**			
0	512 (62)	469 (57)	437 (53)
1	289 (35)	310 (38)	345 (42)
2	13 (1.6)	21 (2.6)	25 (3)
3+	2 (0.2)	18 (2.2)	14 (1.7)
Missing	5 (0.6)	3 (0.4)	0 (0)
**Any changes to where or with whom you live since your last visit**			
Yes	n/a	32 (3.9)	24 (2.9)
No	n/a	785 (96)	797 (97)
Missing	n/a	4 (0.5)	0 (0)
**Risk status**			
Low risk	332 (41)	258 (32)	233 (28)
At risk	489 (60)	563 (69)	588 (72)

^a^Values >10 were rounded to the nearest whole percentage

^b^i.e.*,* sexual contact

**Table 3:** Meningococcal vaccination history among participants present for all three visits; (n = 821).

At least one dose of meningococcal vaccine	All 3 swabs (N = 821) No. (%)
MenACWY vaccine	611 (74)
MenB vaccine Either or both (MenB and/or MenACWY)	299 (36) 624 (76)
Unknown vaccine	5 (<1)

Meningococcal carriage rates across the population increased from 3.5% at baseline (visit 1) to 3.9% at 1 month (visit 2), and 5.7% at 3 months (visit 3). A 63% increase in carriage was observed from visit 1 to visit 3. Among 34 students who had meningococcal carriage detected at visit 1 and presented for visit 2, 20 (59%) maintained carriage of the same meningococcal strain from visit 1 to visit 2, 4 (12%) students acquired carriage of a different meningococcal strain and 10 (29%) were considered to have lost carriage as no meningococcal isolate was recovered. Among the 32 students who had meningococcal carriage detected at visit 2 and presented for visit 3, 16 (50%) maintained carriage of the same meningococcal strain, 2 (6%) acquired carriage of a different meningococcal strain, and 14 (44%) were considered to have lost carriage. Of the students who did not have meningococcal carriage at visit 1, 12/892 (1.3%) acquired meningococcal carriage at visit 2, and of those who were not carrying at visit 2, 29/789 (3.7%) acquired carriage at visit 3 (Table 4).

**Table 4:** Changes in the *Neisserria meningitidis* carriage status between visits among participants.

Visits	Previous visit positive and returned for next visit (n)	Maintained any carriage n (%)	Maintained same carriage n (%)	Acquired different carriage n (%)	Carriage lost n (%)	Previous visit negative and returned for next visit (n)	Acquired carriage n (%)
**All Participants**							
Visit 1–2	**34**	24 (71)	20 (59)	4 (12)	10 (29)	**892** ^a^	12 (1.3)
Visit 2–3	**32**	18 (56)	16 (50)	2 (6)	14 (44)	**789** ^b^	29 (3.7)
**Participants with all three swabs**							
Visit 1–2	**28**	20 (71)	19 (68)	1 (4)	8 (29)	**793** ^c^	12 (1.5)
Visit 2–3	**32**	18 (56)	16 (50)	2 (6)	14 (44)	**789** ^b^	29 (3.7)

^a^– 4 samples positive for *Neisseria lactamica*

^b^– 3 samples positive for *Neisseria lactamica* and 1 positive for *Neisseria mucosa*

^c^– 2 samples positive for *Neisseria lactamica*

A total of 120 meningococcal isolates were recovered during the study. Capsule null and genogroup B meningococci predominated, accounting for 57 (48%) and 46 (38%) isolates, respectively. No genogroup A, C, W or Y meningococcal strains were isolated during the study and a small number of genogroup E and Z isolates was recovered (Table 5).

**Table 5:** The prevalence of meningococcal carriage by genogroup among all participants and participants present at all three visits.

		All Participants				Participants present at all three visits			
Genogroup		All visits (n = 2798)	Visit 1 (n = 1047)	Visit 2 (n = 926)	Visit 3 (n = 825)	All visits (n = 2463)	Visit 1 (n = 821)	Visit 2 (n = 821)	Visit 3 (n = 821)
**ACWY**	n	0	0	0	0	0	0	0	0
**B**	n	46	12	13	21	42	9	12	21
	% of isolate	38	32	36	45	39	32	38	45
	% of carriage	1.6	1.1	1.4	2.5	4.3	1.1	1.4	2.5
**E**	n	14	3	3	8	12	1	3	8
	% of isolate	12	8.1	8.3	17	39	3.6	9.4	17
	% of carriage	0.5	0.3	0.3	1	4.3	0.3	0.3	1
**Z**	n	3	1	2	0	2	1	1	0
	% of isolate	2.5	2.7	5.6	0	1.9	3.6	3.1	0
	% of carriage	0.1	0.1	0.2	0	4.3	0.1	0.2	0
**cnl**	n	57	21	18	18	51	17	16	18
	% of isolate	48	57	50	38	48	61	50	38
	% of carriage	2	2	0.2	2.2	4.3	2	1.9	2.2

The genogroup B isolates were predominately clonal complexes ST-41/44 complex (24/46) or ST-32 complex (14/46) which collectively accounted for 83% of all the genogroup B isolates. Similarly, most capsule null isolates were from two clonal complexes, ST-32 complex (31/57) and ST-198 complex (16/57). Only 5/46 (11%) of the genogroup B isolates were identified as expressing a capsule, including all three ST-4821 complex isolates and two ST-41/44 complex isolates (Table 6).

**Table 6:** Clonal complexes of the recovered meningococcal isolates.

Clonal Complex	Genogroup B (serogroup B^a^)	cnl	Genogroup E	Genogroup Z
ST-32 complex	14 (0)	31	0	0
ST-35 complex	2 (0)	2	0	0
ST-41/44 complex	24 (2)	2	0	0
ST-4821 complex	3 (3)	0	0	0
ST-750 complex	2 (0)	0	0	0
unassigned	1 (0)	0	0	3
ST-1117 complex	0	1	0	0
ST-1136 complex	0	1	0	0
ST-162 complex	0	2	0	0
ST-198 complex	0	16	0	0
ST-269 complex	0	1	0	0
ST-60 complex	0	1	9	0
ST-1157 complex	0	0	5	0

^a^capsular expression detected *in vitro*

ST, sequence type

**Fig 2:**
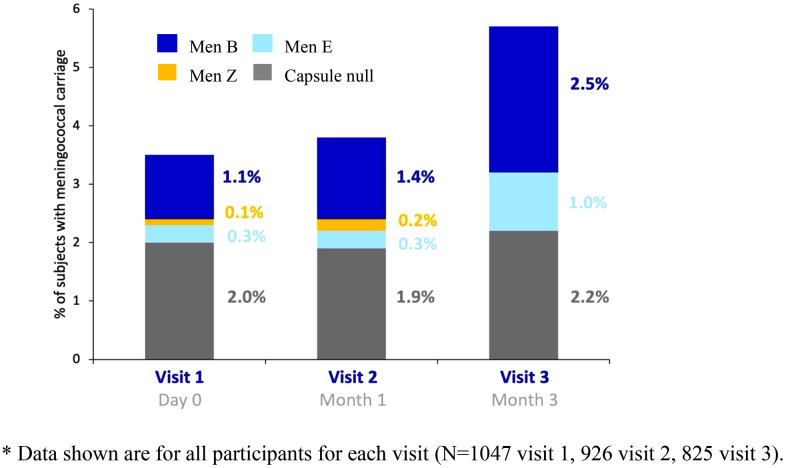
*Neisseria meningitidis* genogroup specific carriage rate at each visit of college students on campus.

Over the three study visits, the carriage rates of capsule null meningococci were relatively similar, as were those for genogroup Z meningococci. Genogroup E carriage increased from 0.3% to 1% between visits 1–3 and genogroup B carriage increased from 1.1% at visit 1, to 1.4% at visit 2 and subsequently became the predominant genogroup at visit 3 with carriage detected in 2.5% of students. (Fig 2)

Among those who presented to all three visits, the visit 1 results (n = 821) had similar independently associated risk factors for meningococcal carriage compared to results from all three visits (n = 2463 swabs). Risk factors included second year vs first year students, living on campus for the second time vs the first time, the type of campus-affiliated housing (apartment vs dorm), as well as smoking tobacco, vaping, kissing and having sexual contact. Ten risk factors were independently associated with meningococcal carriage among those who presented for all three visits (Table 7), however only two (vaping P = 0.023 and party attendance P < 0.0001) remained statistically significant after adjustment (Table 8).

**Table 7:** Risk factors for meningococcal carriage prevalence in a bivariate analysis.

	Visit 1^a^				Visits 1, 2 and 3			
Risk factors	Positives^b^	Negatives	Odds Ratio^c^ (95% CI)	P-value	Positives^b^	Negatives	Odds Ratio^c^ (95% CI)	P-value
**Age (years)**	28	793	1.06 (0.88, 1.27)	0.5393	107	2356	0.97 (0.84, 1.11)	0.629
**Sex** ^**d**^								
Male (reference category)	10	319	1.00	–	36	951	1.00	–
Female	18	473	1.19 (0.55, 2.57)	0.6603	71	1402	1.33 (0.88, 2)	0.1712
**Race**								
Asian (reference category)	0	47	1.00	–	2	139	1.00	–
African American	1	135	1.05 (0.04, 27.02)	0.9757	6	402	0.9 (0.21, 3.94)	0.8900
White	27	525	4.97 (0.29, 85.26)	0.2687	96	1560	3.45 (0.97, 12.31)	0.0563
Other/Mixed	2	86	0.55 (0.01, 29.05)	0.7674	3	255	0.76 (0.15 3.94)	0.7482
**Ethnicity**								
Hispanic (reference category)	2	49	1.00	–	3	150	1.00	–
Non-Hispanic	25	693	0.73 (0.19, 2.79)	0.6428	98	2056	2.06 (0.7, 6.08)	0.1907
Not specific	1	51	0.58 (0.07, 4.62)	0.6039	6	150	1.86 (0.49, 6.97)	0.3592
**Number of roommates**	28	793	1.01 (0.97, 1.05)	0.7263	107	2356	1.01 (0.98, 1.03)	0.638
**Student Status**								
1st Year (reference category)	11	586	1.00	–	59	1732	1.00	–
2nd Year	14	136	5.42 (2.44, 12.03)	<.0001	41	409	2.95 (1.96, 4.45)	<.0001
3rd Year	2	47	2.68 (0.65, 11.01)	0.1703	5	142	1.12 (0.46, 2.74)	0.7977
4th Year	1	18	4.14 (0.68, 25.12)	0.123	2	55	1.31 (0.36, 4.83)	0.6835
5th Year or higher	0	6	3.92 (0.17, 92.59)	0.3966	0	18	0.79 (0.04, 14.27)	0.8713
**Living on campus housing**								
First time (reference category)	11	608	1.00	–	59	1798	1.00	–
Second time	14	134	5.7 (2.57, 12.67)	<.0001	41	403	3.11 (2.06, 4.69)	<.0001
Third time	2	40	3.27 (0.79, 13.49)	0.1019	5	121	1.37 (0.56, 3.35)	0.4927
Other	1	11	6.9 (1.07, 44.54)	0.0423	2	34	2.19 (0.58, 8.26)	0.2468
**Residential situation**								
Dorm (reference category)	15	620	1.00	–	70	1835	1.00	–
Non-dorm	13	173	3.11 (1.47, 6.6)	0.003	37	521	1.87 (1.24, 2.82)	0.0026
**Other cohort identification**								
Member of sport team (reference category)	2	23	1.00	–	4	71	1.00	–
Member of club team	2	39	0.6 (0.09, 3.8)	0.5835	5	118	0.74 (0.2, 2.67)	0.6428
Member of the Band	0	37	0.13 (0.01, 2.85)	0.1928	2	109	0.36 (0.07, 1.77)	0.2093
Not applicable	18	587	0.3 (0.07, 1.21)	0.0902	73	1742	0.67 (0.25, 1.8)	0.4268
**Behavioral risk**								
**Having a cold, sore throat, or respiratory infection**								
No (reference category)	27	770	1.00	–	94	2144	1.00	–
Yes	1	21	1.95 (0.34, 11.08)	0.4494	13	207	1.48 (0.82, 2.66)	0.1948
**Antibiotic Intake**								
None in the last month (reference category)	28	710	1.00	–	98	2063	1.00	–
Stopped in last month	0	38	0.32 (0.02, 5.6)	0.4382	4	113	0.84 (0.32, 2.21)	0.7249
Stopped in last week	0	13	0.92 (0.05, 17.7)	0.9578	3	69	1.12 (0.37, 3.35)	0.8414
Yes, currently taking	0	30	0.41 (0.02, 7.17)	0.5404	2	106	0.34 (0.07, 1.74)	0.196
**Smoked tobacco/cigarettes in the last week**								
No (reference category)	25	763	1.00	–	89	2227	1.00	–
Yes	3	28	3.68 (1.11, 12.15)	0.0327	18	123	3.73 (2.19, 6.36)	<.0001
**Smoked nicotine e-cigarette (vape) in the last week**								
No (reference category)	18	684	1.00	–	67	1973	1.00	–
Yes	10	107	3.61 (1.65, 7.93)	0.0014	40	378	3.13 (2.09, 4.69)	<.0001
**Smoked pot (marijuana, weed, grass, etc.** ^**e**^ **) in the last week**								
No (reference category)	24	691	1.00	–	77	1964	1.00	–
Yes	4	99	1.28 (0.46, 3.58)	0.6426	30	386	2 (1.3, 3.09)	0.0017
**If yes, how?**								
Vape (reference category)	3	35	1.00	–	8	138	1.00	–
Other ^d^	1	64	0.24 (0.03, 1.7)	0.1516	22	248	1.48 (0.65, 3.34)	0.3519
**Smoked by sharing a device in the last week**								
No (reference category)	26	760	1.00	–	98	2216	1.00	–
Yes	2	31	2.28 (0.58, 8.9)	0.2371	9	135	1.58 (0.79, 3.15)	0.1955
**Been to a party in the last week**								
No (reference category)	12	626	1.00	–	24	1481	1.00	–
Yes	16	165	5 (2.35, 10.64)	<.0001	83	870	5.8 (3.67, 9.17)	<.0001
**Kissed or hooked up**^f^ **within the last week**								
No (reference category)	12	500	1.00	–	41	1377	1.00	–
Yes	15	289	2.14 (1, 4.58)	0.0492	65	972	2.24 (1.5, 3.33)	0.0001
**Risk status**								
Low risk (reference category)	3	364	1.00	–	9	849	1.00	–
At risk	25	429	6.18 (2, 19.09)	0.0015	98	1507	5.84 (2.99, 11.43)	<.0001
**Vaccination status**								
Vaccinated (reference category)	19	609	1.00	–	71	1813	1.00	–
Not vaccinated	9	184	1.61 (0.73, 3.56)	0.2404	36	543	1.7 (1.13, 2.57)	0.011

^a^– Among those who presented to all three visits, these are the data from visit 1

^b^– Positive means *Neisseria meningitidis* was identified and Negative means *N. meningitidis* was not identified

^c^– The ORs and the 95% CIs were calculated by exponentiating the estimates obtained from using a bivariate logistic regression model

^d^– sex at birth

^e^– Includes bong, bowl, joint, and other

^f^– *i.e.,* sexual contact

**Table 8:** Risk Factors for Meningococcal Carriage Prevalence in a Multivariable Model.

	Visit 1^a^		Visit 1, 2 and 3	
	Positives^b^ (n = 36), Negatives^b^ (n = 1005)		Positives^b^ (n = 106), Negatives^b^ (n = 2347)	
Risk Factors	Odds Ratio^c^ (95%CI)	P-value	Odds Ratio^c^ (95%CI)	P-value
**Race**				
Asian (reference category)	1.00	–	–	–
Black	1.28 (0.06, 25.52)	0.8731	1.04 (0.24, 4.51)	0.9576
White	5.58 (0.40, 77.36)	0.2001	3.17 (0.89, 11.28)	0.0743
Mixed/Other	2.43 (0.13, 44.18)	0.5481	0.70 (0.14, 3.59)	0.6706
**Student Status**				
1st Year (reference category)	1.00	–	1.00	–
2nd Year	1.61 (0.18, 14.36)	0.6677	0.61 (0.09, 4)	0.6098
3rd Year	1.13 (0.1, 12.43)	0.9202	0.3 (0.03, 2.85)	0.2957
4th Year	0.98 (0.06, 15.37)	0.9888	0.46 (0.04, 5.62)	0.5395
5th Year or higher	2.24 (0.08, 59.48)	0.6287	0.25 (0.01, 10.35)	0.4659
**Living on campus housing**				
First time campus (reference category)	1.00	–	1.00	–
Second time	4.66 (0.53, 40.96)	0.1655	5.96 (0.93, 38.21)	0.0597
Third time	5.58 (0.48, 65.09)	0.1698	4.69 (0.48, 46.01)	0.1844
Other	7.31 (0.53, 100.71)	0.137	6.72 (0.56, 81.11)	0.134
**Residential situation**				
Dorm (reference category)	1.00	–	1.00	–
Non-dorm	0.56 (0.23, 1.35)	0.1947	0.64 (0.33, 1.21)	0.1673
**Smoked tobacco/cigarettes in the last week**				
No (reference category)	1	–	1.00	–
Yes	2.15 (0.69, 6.68)	0.1873	1.72 (0.94, 3.15)	0.0809
**Smoked nicotine e-cigarette (vape) in the last week**				
No (reference category)	1.00	–	1.00	–
Yes	2.69 (1.2, 6.02)	0.0164	1.77 (1.09, 2.87)	0.0203
**Smoked pot (marijuana, weed, grass, etc.) in the last week**				
No (reference category)	1.00	–	1.00	–
Yes	0.44 (0.15, 1.28)	0.1324	0.9 (0.54, 1.51)	0.6887
**Been to a party in the last week**				
No (reference category)	1.00	–	1.00	–
Yes	2.66 (1.37, 5.15)	0.0038	4.71 (2.95, 7.51)	<.0001
**Kissed or hooked up**^**d**^ **within the last week**				
No (reference category)	1.00	–	1.00	–
Yes	0.99 (0.51, 1.91)	0.9713	1.33 (0.88, 2.02)	0.1765
**Vaccination status**			1.00	–
Vaccinated (reference category)	1.00	–	1.72 (0.94, 3.15)	0.0809
Not vaccinated	1.6 (0.81, 3.16)	0.1725		

^a^– Among those who presented to all three visits, these are the data from visit 1.

^b^– Positive means *Neisseria meningitidis* was identified and Negative means *N. meningitidis* was not identified

^c^– The ORs and the 95% CIs were calculated by exponentiating the estimates obtained from using a multivariable logistic regression model

^d^– *i.e.,* sexual contact

## Discussion

Overall, meningococcal carriage was relatively low on arrival to college (3.5%) but increased by 63% (to 5.7%) over the 3-month study period in the semester. Genogroup B was responsible for 39% of meningococcal carriage over the three visits and was responsible for a high proportion of transmission and acquisition. Genogroups ACWY were not detected during the study. Risk factors including smoking tobacco or cannabis, vaping, being a second-year student compared to a first-year student, attending a party and kissing/sexual activity within 1 week of sample collection were found to be significantly associated with meningococcal carriage.

The present study provides the first data on meningococcal carriage in the US since the start of the COVID-19 pandemic. When college students moved in, 37 out of 1047 were colonized (3.5%). This level was lower than other carriage studies previously conducted in academic centers in the US. In 2015, serogroups B, C, W and Y carriage in undergraduates in Rhode Island increased from 12.7% to 14.6% [19]. In 2015−16, serogroup B carriage in students at Oregon University increased from 14% to 17% [20]. This study’s baseline was two months after a new semester started, in contrast to the present study baseline at the beginning of the semester. In 2000, carriage in first year students at the University of Nottingham in Nottingham, England increased from 13.9% to 34%. The “baseline” of all three of these studies was two months *after* the students had been on campus to start a new semester. Two months fell between the second and third visits of the present study when the carriage was between 3.9% and 5.7%, which was substantially lower than their 12.7%, 14.0% and 13.9%, respectively. Overall, the present study had a lower baseline and a smaller increase than previous studies. While there was a 63% increase in carriage over the 12-week period (3.5% to 5.7%), the rate of carriage at each visit underrepresents the true transmission and acquisition events due to students gaining different carriage and students losing carriage between visits.

The initial carriage level found in the present study was also lower than a study performed on campus in Sweden and among new military recruits in Israel. In 2018−19, at Ӧrebro University in Sweden, the initial carriage was 9.5% among 2755 students on both the university’s general and medical campuses [21]. In 2019, Israeli military recruits were enrolled and vaccinated for ACWY on the day of enlistment [22]. Among 639 recruits, 145 (23%) were positive for meningococcal carriage. Both studies found a decrease in carriage over time. The carriage in Swedish students decreased to 8.9% over 12 months. The follow-up was poor, however, with just 11% participating for a second swab, 5% for a third and 1% for a fourth. The carriage in the Israeli recruits decreased to 17% over 8 months. This study had good continuity over time, but it was unclear if the same recruits were being swabbed at subsequent visits. These studies provide a good comparison for an initial carriage value. Other studies that more accurately studied the rates of change were from the UK. Between 1999 and 2015 in students aged 15−19, after the introduction of the MedC vaccine, serogroup B carriage was reported to decrease from 5.6% to 1.8%, serogroup C decreased from 1% to 0.06%, serogroup W decreased from 1.7% to <0.5%, and serogroup Y remained similar at 1.7% to 1.8% [23]. The MenC vaccine was released in the UK in 1999 and given to all children up to 18 years as part of a widespread catch-up campaign. The proportion of students who were vaccinated was not reported, but general data from the UK shows a childhood MenC immunization rate of approximately 90% [24]. At the University of Nottingham, the change in *N. meningitidis* was tested in students over one academic year from the fall semester (September 2015) to the spring semester (March 2016) [25]. Serogroup W increased from 0.7% to 8.0%, serogroup B changed from 3.3% to 5.9% and serogroup Y changed from 1.8% to 2.3%. A study on the same campus in 2008−09 [26], before the emergence of the MenW clonal complex 11 (W:cc11) as a national outbreak strain [27], did not detect any group W.

There are several reasons that may account for the differences in meningococcal carriage at baseline and the rate of increase over time including COVID-19 pandemic restrictions, changes in behavioral habits (exposure risk factors) and the use of meningococcal vaccines. The COVID-19 pandemic prompted significant isolation from the community, and this had a clear impact on reducing disease incidence globally, including *N. meningitidis* [11]. It is possible that reduced meningococcal carriage rates were driven by the number of online classes available, and the proportion of the online/in-person format that classes used. Classroom changes were in effect during the present study (and still are at the time of this writing). How substantially isolation decreased the carriage rate two years later, at the time of the present study, is uncertain. Another reason for differences may be different behaviors. Different cultures have different drinking ages as well, which may have resulted in varying carriage rates among countries (*e.g.,* US versus UK).

Regarding risk factors, the present study confirms that there is still potential risk of disease in students within the college age group compared to other ages [22,23,29–31]. We observed similar factors associated with *N. meningitidis* carriage as prior studies including smoking tobacco [32,33], vaping, close social contact while attending parties [10,21], kissing/sexual contact [22] and history of vaccination. Recent studies have noted a decrease in *N. meningitidis* carriage compared to prior estimates in their population and have suggested this decrease may in part be related to changing social behaviors such as decreased rates of smoking [23]. Few of our participants smoked tobacco (approximately 6% across the study) whereas 15–20% of participants smoked e-cigarettes or marijuana, so approximately one in every five students had the risk factor of sharing smoking paraphernalia (lip-to-device-to-lip exposure). College students are known to consist of the age-group that has the highest meningococcal carriage [28]. More specifically, freshman have been reported to have an increased risk of meningococcal disease [29]. It was unique to the present study, however, that second year students were at a higher risk than first year students. This is an example of how epidemiology changes and why it is relevant to monitor meningococcal carriage should this be found to be generalizable.

The immunogenicity, and ability to prevent carriage, of meningococcal vaccines was studied in 2010 in England among university students aged 18–24 years over one year and categorized into three groups; those who received (i) a meningococcal ACWY vaccine (988), (ii) a meningococcal B vaccine (979) or (iii) a placebo (984) [30]. Both vaccines elicited robust immunogenicity, but neither vaccine reduced carriage. The lack of impact of the MenACWY vaccine in preventing carriage was potentially due to the study design and because affecting carriage requires broader immunization across a larger population.

Historically, one may appreciate an inverse proportion of the influence of meningococcal vaccines on meningococcal carriage rates. For example, in 2017–8, in a cohort of 610 university and high school students in Budapest, Hungary, of whom only 14.1% had been vaccinated, *N. meningitidis* carriage was found in 212 (35%) of the participants [9]; most isolates were non-groupable (87%). While vaccination against serogroup C is recommended in Hungary for infants, adolescents and adults between 14–25 years of age, meningococcal vaccination is not part of the mandatory vaccination program in that country and the high carriage rate there reflects that. On the other hand, a cohort of 421 first-year students at the University of Adelaide in South Australia in 2017, where MenACWY vaccination is part of the country’s national immunization program, the carriage rate of serogroups B, W or Y together was only 6.2% [10]. In addition, carriage was significantly reduced following the introduction of MenC vacccine, and subsequently MenACWY vaccine, to the UK schedule [31]. A similar decrease was noted in the US where rates of genogroup C, W and Y carriage were previously shown to be relatively low in the student population with fewer than 1% carrying any of these genogroups [20,32].

In the present study, the complete absence of genogroup A, C, W and Y carriage in any student in any of the three visits is likely multifactorial, including COVID-19, but may be primarily due to MenACWY vaccination in the US. With 20 years of availability, the generation participating in the present study had the opportunity to acquire the routine MenACWY at 11–12 years and follow-up at 16 years, and subsequently almost three of four students reported receiving MenACWY vaccine [33]. Serogroup C, W and Y meningococcal disease cases still occur in the US, suggesting that carriage is present in other age groups and the population as a whole. The absence of genogroup A, C, W and Y carriage in the present study of college students suggests that there is herd protection, and that without MenACWY vaccination, there would likely be more cases across the population.

Although the overall carriage rates detected in the present study were lower than pre-COVID pandemic studies in the US college students [20], comparison of genotypic data shows a more nuanced picture. Carriage of genogroup B strains at visit 3 in our study was 2.5% and this is comparable to the 2.4% and 2.6% detected in the final visits of the earlier US student studies [19,20]. Similarly, all three studies showed that genogroup B acquisition was the key driver of the increase in overall carriage rates over the study periods and also showed little to no increases in capsule null locus or non-groupable carriage. The explanation of differences in overall carriage between the studies is the prevalence of capsule null locus isolates (our study) or isolates considered non-groupable in the previous studies which included non-genogroup A, B, C, W, X and Y isolates). We detected 1.9-2.2% capsule null locus carriage across the three visits whereas nongroupable carriage was 8−15% (Oregon campus) and 9.9-10.8% (Rhode Island campus), respectively [19,20]. Collectively, these data show that the main difference for the lower overall carriage rate in our study is due to reductions in generally non-pathogenic meningococci. Genogroup B, the genogroup responsible for most disease cases in the student population, remained at the same frequency as 2013−16 when US university outbreaks were occurring, and the previous carriage studies were undertaken. These studies reveal the presence of genogroup B carriage, highlight the lack of herd protection and provide explanation why meningococcal cases occur in this age group. The only way to protect against this is by being immunized in order to gain direct protection from disease.

### Limitations and strengths

This study was limited to one college campus; hence the results may not be generalizable to other populations. There may be selection bias if there were significant differences between participants and non-participants, as only students who signed informed consent were included in the study. Additionally, there may be some recall bias and social desirability bias given the environment of this population and sensitive nature of the questionnaire, which can impact the validity of the results. Social isolation and mask use potentially confounded the carriage results to an unknown degree. Additionally, reduced social interactions along with imposed restrictions and heightened awareness during the pandemic may have affected a student’s decision of participating as well as their exposure risk. Commuters and students living off-campus were not included in the study, but that was intentional to capture students living together. Laboratory detection of *N. meningitidis* relied on culture and did not incorporate PCR as used in some previous carriage studies to improve detection. This culture-based approach may therefore underestimate the true prevalence of carriage, as PCR is known to be more sensitive. Finally, a follow-up questionnaire was not administered to compare to the questionnaires obtained at each visit. Future studies could increase the sample size, add other institutions and even explore other risk factors.

To our knowledge, the present study provides the only meningococcal carriage rates and data on transmission as well as acquisition in the US since the start of the COVID-19 pandemic. We were able to achieve high retention rates: 89% between visit 1 and 2 and between visit 2 and 3 and an overall retention rate of 79% from visit 1 to visit 3. The use of REDCap to electronically distribute and implement the questionnaire at each visit may have increased the completion rate. Another important strength is the choice of methods including culture and whole genome sequencing to identify *N. meningitidis* and fully characterize the genogroups for all isolates.

## Conclusion

In conclusion, this meningococcal carriage study provides valuable insights into the prevalence, distribution, transmission dynamics and genetic diversity of *N. meningitidis* within a college population. Carriage rates of capsule null and genogroup A, C, W and Y meningococci were lower than previous US campus studies undertaken prior to the COVID pandemic. While the pandemic may have been a factor, the likely primary explanation for reduced genogroup A, C, W and Y carriage was the uptake of MenACWY immunization in this student population. Genogroup B carriage, contrastingly, more than doubled across the study visits and became the predominant serogroup at the final visit highlighting the transmission and acquisition events over the semester. These genogroup B carriage rates, and increase over the study, were almost identical to the pre-COVID campus studies in the US; which were undertaken in US universities during serogroup B outbreaks. This highlights the ongoing risk of exposure to genogroup B strains at US universities and provides an insight to why serogroup B disease continues to occur in this demographic and why vaccination against serogroup B disease is important. Risk factors associated with carriage were expected (smoking tobacco and social mixing like attending a party and kissing/sexual contact) and unexpected (second year student), which offer a clearer understanding of transmission dynamics. Future carriage studies should be performed periodically to track epidemiological changes of meningococcus to guide vaccination strategies and public health efforts reducing carriage rates and preventing future outbreaks.

## Supporting information

S1 File Appendix. (DOCX)
