# Supplementary material for: Meningococcal carriage and transmission dynamics in college students in Louisville, Kentucky
Source: PLoS One. 2026 Mar 5;21(3):e0344194. doi: 10.1371/journal.pone.0344194 (PMC12962471; doi:10.1371/journal.pone.0344194)
Supplement: S1 File — (DOCX) [file pone.0344194.s001.docx]

**Appendix A**

**CERID Study Group**

Adil Khan, MD, Aditya Bamboria, MD, Ahmed Abdelhaleem, MD, Aisha Olanike Adigun, MD, Aleena Naeem, MD, Alex Glynn, MA, Anuradha Raut, MD, Arushee Bhatnagar, MD, Baian Khanjar, BSBA (CIS), Brigid Connelly, MS, Deekshitha Turaka, MD, Dooa Said Tayel Mahmoud, MD, Emily Adkins, MPH, Holly Aliesky, BSc Hons, Hector Ventura Banegas, MD, Henry Nabeta, MD, PhD, Ihuoma Esther Ogbuokiri, MD, Jafir Wakeel, MD, Javier Gonzalez Oconor, MD, Jennifer Wick, MLT (ASCP), Karen Garth, MT (ASCP), Keerthi Poladi, MD, Laura Schindler, MT (ASCP), Lucia Puga Sanchez, MD, Matthew Grassman, BSBA (CIS), Muhammad Ali Akhtar, MD, Prathyusha Mudduluru, MD, Rana Usman Anwar, MD, Rayamajhi Niguma, MD, Saad Ahmad, MD, Saad Asghar, MD, Sabrena Garr, MT (ASCP), Shameera Shaik Masthan, MD, Shivam Gulati, MD, Srushan Shankara Bhaktula, MD, Sudeep Chapagain, MD, Tabetha Schultz, BSc, Taylor Gilbert, BSc, Tessa Chilton, MBA, Yusra Irshad, MD
